# The M5nr: a novel non-redundant database containing protein sequences and annotations from multiple sources and associated tools

**DOI:** 10.1186/1471-2105-13-141

**Published:** 2012-06-21

**Authors:** Andreas Wilke, Travis Harrison, Jared Wilkening, Dawn Field, Elizabeth M Glass, Nikos Kyrpides, Konstantinos Mavrommatis, Folker Meyer

**Affiliations:** 1 Mathematics and Computer Science Division, Argonne National Laboratory, 9700 S. Cass Ave., Argonne, IL, 60439, USA; 2 Computation Institute, University of Chicago, 5735 South Ellis Avenue, Chicago, IL, 60637, USA; 3 Centre for Ecology & Hydrology, Maclean Building, Crowmarsh Gifford, Wallingford, Oxfordshire, United Kingdom; 4 Department of Energy Joint Genome Institute, Walnut Creek, CA, USA; 5 Institute for Genomics and Systems Biology, 900 East 57th Street, Chicago, IL, 60637, USA

## Abstract

**Background:**

Computing of sequence similarity results is becoming a limiting factor in metagenome analysis. Sequence similarity search results encoded in an open, exchangeable format have the potential to limit the needs for computational reanalysis of these data sets. A prerequisite for sharing of similarity results is a common reference.

**Description:**

We introduce a mechanism for automatically maintaining a comprehensive, non-redundant protein database and for creating a quarterly release of this resource. In addition, we present tools for translating similarity searches into many annotation namespaces, e.g. KEGG or NCBI's GenBank.

**Conclusions:**

The data and tools we present allow the creation of multiple result sets using a single computation, permitting computational results to be shared between groups for large sequence data sets.

## Background

Similarity searches are potentially the most widely used type of sequence analysis. In some research projects, namely metagenomics [1], the computational costs of similarity searching rapidly outstrips the cost of sequencing [2], [3]. Widely used metagenome analysis systems like IMG/M[4] and MG-RAST[5] employ substantial computational resources while computing sequence similarity results.

Replacing the algorithms used to perform similarity searches (most commonly BLAST [6]) with more efficient algorithms like BLAT[7] will provide a much needed reduction in analysis cost. However, with comparative reference databases growing rapidly, a valuable addition to metagenomic analysis would be the ability to compute similarity searches only once and then exchange the results. Adoption of a needed common reference is currently complicated by the fact that NCBI's non-redundant protein database (“nr”) captures only a single annotation. All (in INSDC parlance) third-party annotations remain excluded. Researchers interested in enzyme numbers (ECs) or SEED subsystem identifiers are forced to repeat similarity searches (against the same body of proteins). Further, most genome (e.g. KEGG [8], SEED [9], IMG [5]) and protein family (eg. KEGG Orthologs [10], SEED FIGfams [11], COGs [12] or EGGnog [13]) (re-) annotation efforts are not captured by NCBI's nr.

Many groups have created sequence identifier-based mechanisms for cross-linking database annotations (see e.g. [14] or [15]). However, these are provided via web-based services and do not lend themselves to efficient local queries at the rates of several hundred thousand identifiers per second that are used by systems like MG-RAST to “translate” large numbers of sequences from one “namespace” into another. One example of this cross-linking is the ability to map the abundance of metagenomic reads onto COG categories, and then compare these to the same reads mapped onto SEED subsystems. With multiple groups offering re-annotation of complete genomes, protein families, or analyses of metagenomes, an efficient way to reduce the overall resource consumption is by using a collapsed sequence database. This approach will allow multiple interpretations of a single similarity search rather than having to run separate searches against various individual databases. We anticipate more adopters of this resource and approach, like IMG/M.

## Construction and content

We have developed a non-redundant protein database (MD5nr) based on the use of MD5 checksums. Our approach separates sequence data from metadata as the sequence and annotation data made available by different groups can logically be split into sequence data and metadata. The data are the raw sequences. The metadata contain sequence identifiers, potential species identifiers, and annotations. Annotation can exist in many forms, including a) free text (e.g. GenBank), b) mappings onto carefully curated functional namespaces (e.g. SEED), and c) mapping onto abstract protein families mixed with free text (e.g. COGs). In addition to storing identifiers, functional annotations and taxonomic information (e.g NCBI taxonomy IDs), we also store mappings to functional hierarchies including GO [16], KEGG orthologs and pathways [17], SEED Subsystems [9], eggNOGs and NCBI COGs.

To create a sequence identifier, we use a 32 character hexadecimal MD5 [18] fingerprint and link all other metadata to this “MD5 ID” [19,20]. Based on this principle, we built a framework suited to the automatic maintenance of a comprehensive, non-redundant protein database. The framework supports the importing of additional protein databases with third party annotations, thereby creating new “namespaces.” Adding another set of annotations appends the metadata only, not the sequence data set, making this approach scale-able. For each additional data source, a mapping of MD5 IDs to annotations is included.

**Figure 1:**
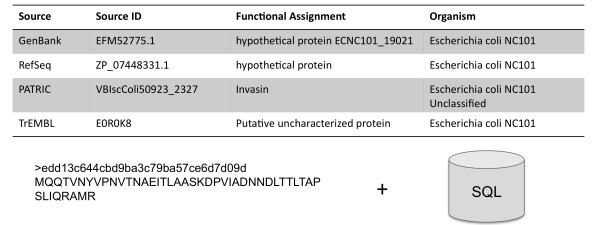
**A simplified view of the internal representation of the M5nr.** Sequences are stored in a single FASTA file using md5 sequence identifiers. In addition a number of tables are stored in an SQL database management system to allow rapid queries. The tables link md5 identifiers with IDs, functions and organisms provided by a number of data sources.

The sequence data with MD5 IDs is provided as a simple FASTA file with the indices required for BLAST and BLAT analysis. Metadata from all namespaces is provided in tabular format and in SQL format. The simple SQL schema employed allows efficient queries, optimized for allowing the translation of thousands of terms from one namespace into another using the MD5 identifiers (Figure 1) in seconds. While we provide an instance of the ID resolution service, we would prefer users to install a local copy of the ID resolution service as that offers improved performance.

### The M5nr database and tools

The result of our work is the non-redundant M5nr database and a suite of tools for using and maintaining it. Among the tools is a database schema representing the metadata and Perl and Python scripts for querying the data. We will provide automated quarterly updates of the M5nr. The software and schema are available as a simple tar file for local installation on the M5nr web site. In addition to this we also make all files involved in creating the database available.

**Figure 2:**
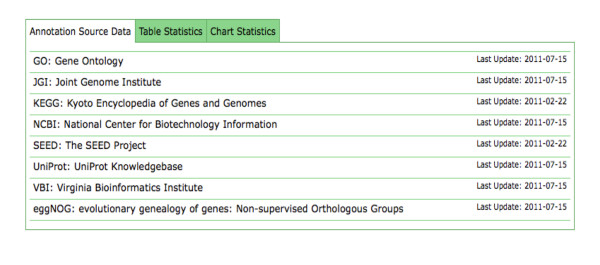
**M5nr Databases.** Databases currently included in the M5nr database as presented in the online overview page provided as part of the M5nr web site.

The comprehensive database already includes a significant number of the available data sources (see Figure 2). In addition to the protein version of the database, we also provide a ribosomal database that has similar properties.

**Figure 3:**
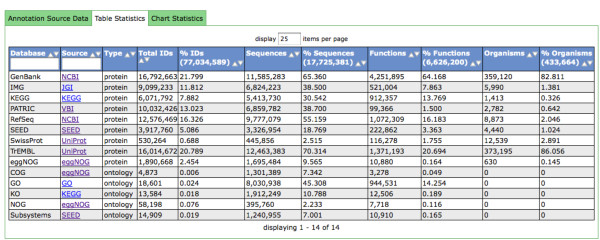
**Database Statistics.** Statistics on the M5nr databases showing total number of source databases, IDs, sequences and other key annotations. We show the number of unique elements added by each database that is added to M5nr. Looking at identifiers, sequences, functional annotations and organisms. For each item (IDs, sequences, functions, organisms) there is a total and percent. The total represents the count of unique representations of that item. The total count is important because there is duplication of sequences, functional names, and organism names within each source database.

Currently the protein database has 15,945,780 unique proteins and 5,793,086 protein functional annotations from eight sources comprising 14 databases Figure 3. The resulting FASTA file that forms the basis for the similarity computations is 6.7 Gigabytes (GB). Compare this to the size of the NCBI nr of currently 7.6 GB (June 27,2001 version). The difference in file sizes is primarily due to the reduced FASTA headers in the M5nr. Overall, the two databases have a similar number of unique sequences.

Using the CLOVR [21] virtual machine environment, we present two use cases that highlight the utility of the system:

#### Use Case 1

The first use case examines the use of a simple API that was developed to allow users the ability to query the database. The following example demonstrates an inquiry of a translation of MD5 IDs to NCBI COG similarities.

Our simple API code:

use M5NR;

use M5NR_Config;

my $md5s = [‘068792e95e38032059ba7d9c26c1be78’];

my $M5nr = M5NR- > new();

my $data = $M5nr- > md5s2sets4source($md5s, ‘COG’);

Users can mine the database through this API using simple command line queries such as:

>m5tool -md5 068792e95e38032059ba7d9c26c1be78 -option md52overview -source COG

where 068792e95e38032059ba7d9c26c1be78 is the md5 ID and COG is the desired annotation source.

This example returns a list of COG functions for the MD5 ID. Users can query any number of IDs at once. More detailed information on API command line arguments can be found at http://blog.metagenomics.anl.gov/m5tools-pl-the-m5nr-database-command-line-tool/.

#### Use Case 2

BLAST or BLAT results against the M5nr in tab format can be mapped to functions and organisms using the command line tool:

>m5tool -sims BLAST_SIMILARITIES -source COG

#### Use Case 3

In order to show the differences in computational cost for performing similarity searches against the M5nr versus individual databases, we BLASTed the MetaHIT metagenome (2.3 Gbps) against M5nr and UniProt (11,256,491 seqs), GenBank (10,232,124 seqs), SEED (3,918,079 seqs), and KEGG (5,413,730 seqs) individually (the version of the MetaHIT metagenome used is MG-RAST ID = 4448044.3). This test used default BLAST parameters. Specifically, for the test, parameter optimizations were not used. All times listed are local executing times measured using the unix time() command. Searching against four of the individual databases took 2935.2, cpu-hours (1072 UniProt; 974.5 GenBank; 373.1 SEED, and 515.6 KEGG) while searching against the M5nr took considerably less time at 1518.6 cpu-hours. If a user wanted to also view additional annotations from other sources, they would have to run those separately, thus increasing the overall compute time. The M5nr provides annotations for all four sources in a fraction of the time. M5nr provides a convenient mechanism to translate annotations from source to another with only one compute.

The M5nr provides sequence files via FTP for use on the users local blast/blat installation. BLAST or BLAT searching via the web site is not provided. MG-RAST uses the M5nr, so users have the option of running their metagenomic sequences against the latest version there.

## Utility and discussion

DNA sequencing is moving from an activity performed at a few centers to a widespread, democratized, and decentralized activity. During the time of this paradigm shift, we are seeing annual increases in throughput by a factor of 10. This growth imposes new requirements on the bioinformatics community providing sequence analysis tools. Enabling the exchange of sequence similarity searches to limit computational costs must be a cornerstone of the new paradigm's foundation. With a standard encoding and searches executed against a standardized database, tools like MG-RAST or IMG/M can now allow users to download large sequence sets and display them in the other analysis and visualization tools, without the need for massive re-computation.

The work presented here is part of the roadmap laid out by the GSC's M5 project (see http://gensc.org) that aims at creating a platform for ubiquitous exchange of computed results for metagenomes along with primary data and metadata. We encourage not only the use of the M5nr but also invite the contribution of alternate annotations. In addition we invite interested developers to contact the GSC's M5 group if they plan to provide additional functionality.

## Conclusions

We present one cornerstone that has the potential to enable large scale sharing of sequence data accompanied by similarity results. The new technology provides mapping of similarity results onto almost arbitrary namespaces by the data consumer in very little time, allowing, for example, user interfaces that show multiple interpretations of data. With the added flexibility, we anticipate that the M5nr can help reduce the computational cost of doing metagenomics significantly.

## Availability and requirements

All software used to create this database is open source and available on the web site under an open source license. The software is intended to run in the CLOVR virtual machine environment, but it can also be downloaded as a stand-alone package. We maintain a reference instance of the system and provide reference quarterly releases of the M5nr. Releases of the M5nr can be found at ftp://ftp.metagenomics.anl.gov/data/M5nr/current/M5nr.gz. The scripts used to generate the M5nr are available on the M5nr FTP site ftp://ftp.metagenomics.anl.gov/data/MD5nr/code/ and github (https://github.com/MG-RAST/M5nr).

We use a purposefully simple shell script to download all required databases to a local repository. After downloading the sequence and annotation data, it is then converted from a number of source formats into an internal format; this step creates the MD5 checksum. Once converted, all databases are merged in the third and final step. Since we chose to match by sequence identity, the time used for creating the M5nr is spent mostly on downloading (typically up to 24 hrs) and less on computing (12 hours on a single core).

## Abbreviations

API, Application programming interface; BLAST, Basic local alignment search tool; BLAT, BLAST-like alignment tool; CLOVR, The cloud virtual resource; COG, Clusters of orthologous groups; GSC, Genome standards consortium; IMG/M, Integrated microbial genomes/Metagenomes system; INSDC, The international nucleotide sequence database collaboration; KEGG, Kyoto encyclopedia of genes and genomes; M5, Metagenomics, Metadata, MetaAnalysis, Models and MetaInfrastructure; MD5, Message-digest algorithm; MG-RAST, Metagenomics RAST (Rapid Annotation using SEED Technology); NCBI, National center for biotechnology information; SQL, Structured query language; EC, Enzyme commission number.

## Competing interest

The author(s) declare that they have no competing interests.

## Authors’ contributions

AW led the overall design and implementation. All other authors participated equally to the development of the resource and writing of the paper. All authors read and approved the final manuscript.
